# Mining gene expression data for rational identification of novel drug targets and vaccine candidates against the cattle tick, *Rhipicephalus microplus*

**DOI:** 10.1007/s10493-023-00838-8

**Published:** 2023-09-27

**Authors:** Christine Maritz-Olivier, Mariëtte Ferreira, Nicholas A. Olivier, Jan Crafford, Christian Stutzer

**Affiliations:** 1 Department of Biochemistry, Genetics and Microbiology, Faculty of Natural and Agricultural Sciences, University of Pretoria, Pretoria, Gauteng, South Africa; 2 DNA Microarray Laboratory, Department of Plant Sciences, Faculty of Natural and Agricultural Sciences, University of Pretoria, Pretoria, Gauteng, South Africa; 3 Department of Veterinary Tropical Diseases, Faculty of Veterinary Science, University of Pretoria, Pretoria, Gauteng, South Africa

**Keywords:** *Rhipicephalus microplus*, Bm86, Multicomponent vaccine, DNA microarray, Antigen selection, Drug target identification

## Abstract

Control of complex parasites via vaccination remains challenging, with the current combination of vaccines and small drugs remaining the choice for an integrated control strategy. Studies conducted to date, are providing evidence that multicomponent vaccines will be needed for the development of protective vaccines against endo- and ectoparasites, though multicomponent vaccines require an in-depth understanding of parasite biology which remains insufficient for ticks. With the rapid development and spread of acaricide resistance in ticks, new targets for acaricide development also remains to be identified, along with novel targets that can be exploited for the design of lead compounds. In this study, we analysed the differential gene expression of *Rhipicephalus microplus* ticks that were fed on cattle vaccinated with a multi-component vaccine (Bm86 and 3 putative Bm86-binding proteins). The data was scrutinised for the identification of vaccine targets, small drug targets and novel pathways that can be evaluated in future studies. Limitations associated with targeting novel proteins for vaccine and/or drug design is also discussed and placed into the context of challenges arising when targeting large protein families and intracellular localised proteins. Lastly, this study provide insight into how Bm86-based vaccines may reduce successful uptake and digestion of the bloodmeal and overall tick fecundity.

**Supplementary Information:**

The online version contains supplementary material available at 10.1007/s10493-023-00838-8.

## Introduction

The Asiatic blue tick, *Rhipicephalus microplus* (Canestrini), is considered one of the most successful tick species of cattle worldwide due to its adaptability to various climatic conditions (Nyangiwe et al. 2017), its displacement of native tick species (Coetzer and Tustin 2004; Tønnesen et al. 2004; Muhanguzi et al. 2020; Nyangiwe et al. 2017), rapid development of acaricide resistance (Lovis et al. 2013; Nyangiwe et al. 2013; Rodriguez-Vivas et al. 2018), and its ability to transmit a variety of economically important tick-borne diseases (Pereira et al. 2022). Currently, acaricides form the cornerstone of tick control globally, but with the growing evidence of resistance against all major classes of acaricides (Dzemo et al. 2022), there is an urgent need for complimentary and/or alternative tick control strategies.

Currently high-throughput data is being mined for novel anti-parasitic drug targets, and recent examples include evaluation of: piperazine derivatives against *Leishmania* species (Schadich et al. 2022), acetylcholinesterase inhibitors and targets for *R. microplus* (Cerqueira et al. 2022), as well as transmission blocking drugs against an array of tick-borne diseases (Schorderet-Weber et al. 2018). As more genomic and transcriptomic data for parasites and pathogens of interest become available, the focus has shifted from classical biochemical screening of chemical libraries (Woods and Williams 2007) to in silico screening of targets including: analysis of the physiochemical properties of the protein, structural modelling, high-throughput screening of large compound libraries and drug docking (e.g., *Shigella dysenteriae*) (Jalal et al. 2022). For ticks, however, the lack of protein crystal structures remains a bottleneck, along with the vast amount of uncharacterized tick-specific proteins, hinder high-quality protein modelling. The next challenge remains the bioavailability and delivery of the antiparasitic drug to the correct protein target (Lipinski et al. 1997), as well as the druggability of these targets (Kozakov et al. 2015; Cimermancic et al. 2016; Fauman et al. 2011).

In the pursuit of novel tick control, tick vaccines have been explored as a complementary approach to chemical control (Stutzer et al. 2018). The rationale for the identification and selection of target antigens remains a severe bottleneck in vaccine development. Early conventional methods, such as protein fractionation, followed by immunization and challenge in animal models, have been adapted to more modern ‘genomics to vaccinology’ approaches (i.e., reverse vaccinology). As the number of genomes being sequenced increases, data on associated transcriptomes and encoded proteomes also increases, and therefore data on various tissues over the duration of the parasite lifecycle could be obtained (Maritz-Olivier et al. 2012; Garcia et al. 2020; Tirloni et al. 2020; Stutzer et al. 2013). This provides valuable insight into the kinetics of antigen expression which is a crucial parameter when selecting antigens for vaccines (Moxon et al. 2019). In silico analyses can identify open reading frames used (among others) to predict similarity to other known vaccine antigens, as well as determine protein characteristics such as putative subcellular localization (e.g., surface expression, secretion, etc.) (Moxon et al. 2019). This can be followed by cloning and recombinant protein production of promising antigens, for vaccination and challenge studies, as well as raising specific antibodies for cell culture studies (e.g. expression library immunization or ELI) (Almazán et al. 2003). As the tools and criteria for in silico identification of surface proteins and protective antigens improve, it is continuously shaping the future of rational vaccine design.

Despite the strides in identification of new vaccine candidate antigens, Bm86-based vaccines remain the most effective against *R. microplus* infestations under field conditions to date (Ndawula and Tabor 2020; Pereira et al. 2022). The Bm86 antigen provided the first proof of concept for a purified single antigen tick vaccine (De La Fuente et al. 2007), and currently the Bm86-based vaccines Gavac™ (and Gavac Plus™) (Herber-Biotec S.A., CIGB, Camagüey, Cuba), as well as Bovimune Ixovac (Lapisa, Michoacan, Mexico) (Blecha et al. 2018), are available in Latin America. A better and deeper understanding of host–parasite interactions (e.g. detailed host immune response analyses), as well as parasite biology (i.e., the metabolic pathways and proteins involved, throughout the life cycle), is desperately needed. For *R. microplus*, more than 38,827 putative gene loci encoding around 24,785 putative proteins are contained in the most recent genome on the NCBI database (GCA_013339725.1), and more than half of these proteins are unique to the tick and remain largely unannotated (Barrero et al. 2017). With such a large and repetitive genome and probable variability in transcriptome kinetics (influenced by life stage and environmental factors), it can be hypothesized that multi-stage/multi-component cocktail vaccines (i.e., including more than one antigen) could enhance the level of protection afforded against infestation (Pereira et al. 2022). This appears to be a trend for vaccines against other complex parasites/pathogens, in example vaccines against: helminths (Maizels 2021), endoparasites such as malaria (Pirahmadi et al. 2021), *Theileria* (Atchou et al. 2020; Saaid et al. 2020), *Babesia* (Rathinasamy et al. 2019) and intracellular bacteria (Osterloh 2022), as well as ticks (Pereira et al. 2022; Costa et al. 2021). However, some cocktail vaccines tested against *R. microplus* did not live up to expectations (Pereira et al. 2022), and this is likely due to: antigen type (i.e., full-length, peptide, chimera, etc.) and quality; protein production platform (e.g., bacterial, yeast, etc.); antigen concentration; antigen-adjuvant interaction; antigenic competition; or even animal genetics, where information on the impact on the host immune system is incomplete (Ndawula and Tabor 2020; Stutzer et al. 2018).

In this study, we aimed to characterize the compensatory mechanisms used by *R. microplus* ticks to overcome the deleterious effects of host immunity when feeding on cattle hosts vaccinated with a Bm86-based multicomponent formulation (i.e., containing Bm86 and three putative Bm86-binding proteins, University of Pretoria invention disclosure) using DNA microarrays and subsequent differential gene expression profiling. We wanted to determine whether high-throughput transcriptomics can be used to elucidate biological reasons for why a vaccine can fail to confer sufficient protection (e.g. compensatory mechanisms), as well as whether this data can be used for the rational selection of additional vaccine candidates based on their putative function and accessibility to the host immune system (i.e., extracellular membrane bound or secreted). The data generated in this study was also evaluated, based on published examples, for identification of novel pathways and druggable targets to direct future efforts in next generation chemical control.

## Material and methods

### *Rhipicephalus microplus* tick strain

Pathogen free *Rhipicephalus microplus* larvae, from a laboratory bred strain of South African origin, were obtained from ClinVet International (Bloemfontein, South Africa). Shortly, batches of 8 to 10 engorged females fed on donor cattle are placed into 200 ml conical flasks and allowed to lay eggs under controlled conditions (28 °C, 80% humidity). The resultant larvae are used for infestation trials within 2 months of occlusion.

### Cattle vaccination trial

6- to 8-month-old Holstein–Friesian (*Bos taurus*) female calves were housed at the Onderstepoort Veterinary Animal Research Unit (OVARU), University of Pretoria, Onderstepoort, South Africa. Calves (6–7 months of age) were treated prophylactically, a week ahead of commencement of vaccination studies, with Engemycin 10% 20 mg/kg intramuscularly, Baycox 5% 3 ml/10 kg per os, Valbazen 1 ml/10 kg per os, Berenil 3.5 mg/kg subcutaneously and Kyroligo 5 ml/calve intramuscularly, as well as dipped with Amitraz (Coopers Triatix 12.5%, MSD Animal Health, South Africa), as per manufacturer’s instructions, to remove any ectoparasites prior to commencement of vaccination. The health of the calves was further evaluated by examining peripheral blood smears for haemoparasite screening and haematocrit determination from drawn blood, as well as monitoring calf weights (weekly) and temperatures (daily) for the duration of the study. Ethical clearance was granted for the acquisition and rearing of ticks, as well as to study the effect of vaccination by the University of Pretoria Animal Ethics Committee (Project numbers: EC036-13) and from the Department of Agriculture, Land Reform and Rural Development (Section  20 of the Animal Diseases Act 1984, reference number: 12/11/1/8/1). Cattle were divided into two groups of four individuals (n = 4) at random, i.e., a adjuvant/saline control group and a test group vaccinated with Bm86 in combination with three novel *R. microplus* antigens (i.e., RmAg1, RmAg2 and RmAg3) at 50 µg each. Antigens were formulated with 1:1 (v/v) Montanide ISA 71 VG (Seppic, France) and a final volume of 1 ml was administered subcutaneously in the neck. Recombinant Bm86 was derived from the Mexican susceptible CENAPA strain, Genbank accession number ACR19243, produced in *Pichia pastoris* and kindly supplied by Prof J de la Fuente, Spain. The putative binding proteins (1–3) were produced in *Escherichia coli* (Biologics Corp, USA). A first booster immunisation was administered 4 weeks after the initial immunisation (i.e., Day 28), and a second booster after an additional 14 days (i.e., Day 42). An estimated 4000 larvae were used per animal for full body infestation 10 days after administration of the second booster. Ticks were allowed to complete their life cycle and feed to engorgement. Dropped/fully engorged female ticks were collected, counted, weighed, and placed in small, aerated plastic containers and incubated at 28 °C (80% humidity) in a humidifying incubator for ovipositing. The weight of laid eggs per female was measured.

Tick vaccine efficacy (E) was calculated as $E \left( \% \right) = 100\left( {1 - \mathop \prod \nolimits_{k = 1}^{n} a_{k} } \right)$ where $\mathop \prod \nolimits_{k = 1}^{n} a_{k}$ represents the reduction in the studied developmental processes $(k)$ in ticks fed on vaccinated cattle as compared to the control fed on adjuvant/saline injected cattle (Cunha et al. 2013). The efficacy of the vaccine was calculated considering the effect on the reduction of tick infestations (i.e., log-transformed number of dropped female ticks per animal), feeding efficiency (i.e., the weight of dropped female ticks) and oviposition (i.e., the weight of eggs laid per dropped female tick) as 100 $\left[ {1 - \left( {{\text{CRT }} \times {\text{ CRW }} \times {\text{ CRO}}} \right)} \right]$ where CRT, CRW and CRO are the reduction in the number of female ticks, weight of female ticks and oviposition compared to the control group, respectively.

Data were analysed statistically by a one-tailed Student’s *t* test using a significance level of 0.05. Before determining significance, the Bartlett’s test for homogeneity of variances was performed on all data to test that variance are equal between the control and test groups. Using these results, either a one-tailed Student’s *t* test with equal- or unequal variance was performed. To determine statistical outliers, the interquartile range (IQR) of the number of ticks per group, as well as the first and third quartiles were calculated using Microsoft Excel 2010. The IQR was multiplied by 1.5 and subtracted from the first quartile and added to the third quartile. Data values that fall out of this range were considered statistical outliers.

### Enzyme-linked immunosorbent assay

Blood samples were collected from cattle before commencement of the study (i.e., Day 0), 13 days after the first booster (i.e., Day 42) and 14 days after the second booster (i.e., Day 56). Briefly, 96 well MicroWell™ MaxiSorp™ flat bottom plates (Nunc, Denmark) were coated with 100 ng antigen diluted in TBS (Tris-buffered saline, 25 mM Tris–HCl, 150 mM NaCl, pH 7.5). Plates were dried overnight at room temperature, followed by four washing steps with 350 µl TBS-T (TBS containing 0.05% v/v Tween 20, pH 7.5). Blocking was performed using 350 µl blocking buffer (TBS-T containing 0.5% w/v casein, pH 7.5) for one hour at room temperature, followed by four washing steps. Serum samples were diluted in blocking buffer, added to the plates, incubated for 1 h at room temperature and washed four times. A 1:100 (for RmAg1, RmAg2 and RmAg3) and a 1:4000 (for Bm86) dilutions were prepared from primary serum collected on day 0, 42 and 56. Total bound immunoglobulin antibodies were detected by incubation with 1:6000 (v/v) diluted horseradish peroxidase-conjugated goat α-bovine IgG (Abcam Biotechnology company, UK). Following a final wash cycle, 200 µl substrate solution (2.2 mM o-phenylenediamine dihydrochloride, 0.05 M phosphate-citrate buffer, pH 5, 0.012% fresh H_2_O_2_) were added and the reaction monitored using the Multiskan Plus reader (Thermo Fisher Scientific, USA) at 450 nm with a reference filter at 690 nm. Antibody titres in vaccinated cattle were expressed as the OD_450nm_ (OD_cattle sera_ − OD_baseline_) and compared between the different time points by one-way ANOVA (α = 0.05).

### Isolation of total RNA and cDNA synthesis

Twenty semi-engorged female ticks from each of the four cattle allocated in the combination vaccinated and control (untreated) groups (i.e., 80 ticks per group), respectively, were collected 20 days post infestation. Midgut tissues were collected by dissection and homogenized in TRI Reagent (Sigma-Aldrich, Germany), frozen in liquid nitrogen and stored at −80 °C until RNA isolation was performed. Total RNA isolation, first strand cDNA synthesis, RNA hydrolysis and cDNA purifications were performed as per the methods outlined by Stutzer et al. (2013).

### DNA microarrays

Microarray analysis was performed using a custom 4 × 44 K microarray slide platform designed from the transcriptome data (*Rmi*GI Version 3 Gene Index) derived from the genome assembly of *R. microplus* (Wikel strain). A total of 28 913 sequences were submitted online for array design using the Agilent 4 × 44 k microarray and eArray microarray design platforms (https://earray.chem.agilent.com/earray/). Vector sequences derived from EST data were removed. Using the standard base composition probe design strategy, 60 mer probes representing the full complement of transcripts were designed. As controls, duplicates of half of the transcripts were distributed at random in the array design together with other standard incorporated control probes. A reference pool experimental design was used for transcriptome analysis of *R. microplus* fed on control and vaccinated (i.e., Bm86 and putative binding proteins combinatorial) cattle. Dye coupling via incorporated amoinoallyl dUTPs was achieved by the addition of 2.5 µl of Cyanine 3-dCTP (reference pool) or Cyanine 5-dCTP (test sample) (Amersham Biosciences, UK). Equivalent concentrations of the dye coupled sample and reference pool cDNA (50 pmol each) were hybridised to the microarray slide for 17 h at 65 °C using the Agilent Gene Expression Hybridisation Kit (Agilent Technologies, USA) according to the manufacturer’s recommendations. Following hybridisation, the microarray slides were washed, rinsed, and dried prior to scanning using the Axon GenePix™ 4000B microarray laser scanner (Molecular Devices, USA).

### In silico data analyses and functional annotation of differentially expressed transcripts

DNA microarray data analysis was performed as per the method outlined by Stutzer et al. (2013), using the Robust spline method for within-array normalisation (M values). The threshold for significance was based on adjusted *P* < 0.01 and the upper and lower 1% log_2_FC distribution of differentially expressed transcripts in the vaccinated group relative to the control group. Normalized microarray data was submitted to the GEO repository of the National Centre for Biotechnology Information on accession no GSE221646.

Microarray probe sequences were annotated using the comprehensive annotation suite Blast2GO (http://www.blast2go.com) (Conesa et al. 2005). Additional similarity searches were performed using BLASTX or BLASTP searches against selected databases, i.e., the tick annotation release 100, based on the assembly ASM1333972v1 (GCF_013339725.1) (Jia et al. 2020), the Rmi2.0 annotation based on assembly GCA_002176555.1 (Barrero et al. 2017) and the RefSeq Invertebrate Protein database with sequences containing Acari [organism] protein sequences obtained from GenBank (Mistry et al. 2020).

### Semi-quantitative real-rime PCR validation of array results

To verify the differential gene expression results obtained from DNA microarray analysis, RT-qPCR analysis was performed. Gene specific primers were designed for 10 transcripts that were up- and downregulated, respectively, for vaccinated vs. control group (Supplementary Table 1). Semi-quantitative PCR was performed as per the method outlined by Stutzer et al. (2013), using 10 pmol of oligonucleotide primer pairs corresponding to the selected sequences that were differentially expressed. The mRNA levels were normalised against two reference genes namely, elongation factor I-alpha (ELF1α) and ribosomal protein L4 (RLP4), that were previously validated to have stable expression in *R. microplus* (Nijhof et al. 2009). All reactions were carried out in duplicate. The Quant Studio 12 K-flex system was used for all reactions with its corresponding software (Life Technologies Biotechcompany, USA). Quantitative analysis was performed using qBase^PLUS^ software (Biogazelle, Zwijnaarde, Belgium–http://www.qbaseplus.com) (Hellemans et al. 2007). The relative transcript levels for selected genes were evaluated using the extracted calibrated normalised relative quantities (CNRQ) and expressed as a fold change (on a log_2_ scale) relative to the reference genes selected. These normalised values were used to calculate the fold change for selected transcripts. Analysis of variance (ANOVA) was used to determine significant differential gene expression compared between the test and control groups (α = 0.05).

## Results and discussion

### Bovine antibody response to vaccination

**Fig. 1:**
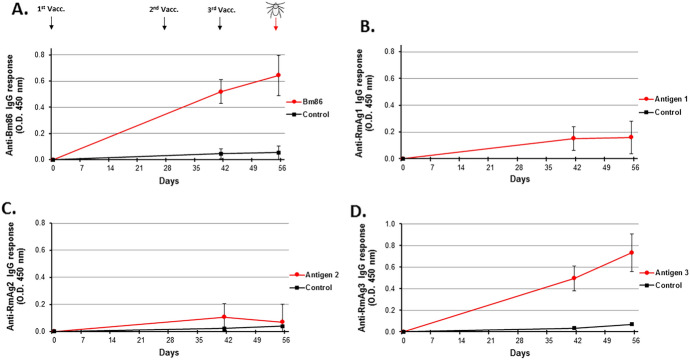
Antigen-specific antibody responses in cattle vaccinated with a four-antigen combinatorial vaccine (**A**–**D**). Indicated is total IgG serum antibody responses raised in vaccinated cattle against recombinant Bm86, RmAg1, RmAg2 and RmAg3 over a period of 2 months. Control animals were injected with a saline buffer solution formulated in Montanide ISA 71 VG adjuvant. Antibody responses are expressed as the OD 450 nm value for using a 1:100 (i.e., RmAg1, RmAg2 and RmAg3) and 1:4000 (i.e., Bm86) dilutions of serum collected at day 0, 42 and 56. Error bars indicate standard deviation of antibody responses between animals of the same group (n = 4), performed with technical replicates. Black arrows indicate the days of vaccination at day 1, day 28 and day 42. Red arrow indicates the day of infestation at day 55 (color figure online)

Indirect ELISA-results demonstrated that no antigen-specific IgG response and cross-reactive responses against Bm86 were observed in control cattle. Cattle from the test group developed an antibody response against Bm86 (Fig. 1A) which increased significantly after the second vaccination (*P* = 1.04E−04) and the third vaccination (*P* = 0.023), before cattle were challenged with larvae on day 55. Weak immune responses were observed against RmAg1 (Fig. 1B) and RmAg2 (Fig. 1C), while a significant response against RmAg3 (*P* = 3.27E−04) was achieved (Fig. 1D).

### Effect of vaccination on *Rhipicephalus microplus* feeding

A 44.7% reduction in the number of engorged female ticks from cattle vaccinated with the combinatorial Bm86 vaccine, compared to the control group, was observed *(P* = 0.033; Table 1). Though the tick- and egg weights were not significantly affected, the calculated protective efficacy of the vaccine was 40.3% (Table 1).

**Table 1:** Effects on females, their weight, oviposition, and efficacy of vaccination with rBm86 (Mexican susceptible strain), RmAg1 to 3 against *Rhipicephalus microplus* South African strain infesting cattle

Group	Cattle number	Number of dropped females	Average dropped female weight (g)	Average egg weight (g)
Control (A)	A1	120	0.24 ± 0.09	0.10 ± 0.04
	A2	113	0.29 ± 0.06	0.11 ± 0.03
	A3	75	0.30 ± 0.10	0.12 ± 0.05
	A4	55	0.28 ± 0.08	0.12 ± 0.07
Average ± SD		90.75 ± 30.97	0.28 ± 0.02	0.12 ± 0.01
Bm86 combi (B)	B1	39	0.27 ± 0.09	0.11 ± 0.03
	B2	51	0.28 ± 0.07	0.13 ± 0.03
	B3	161	0.28 ± 0.09	0.08 ± 0.05
	B4	55	0.28 ± 0.08	0.13 ± 0.04
Average ± SD		48.33 ± 8.33*	0.28 ± 0.00	0.13 ± 0.02
Efficacy^a^ (%)	40.3			

^a^Efficacy (%) = 100 [1 − (CRT × CRW × CR0)]; where CRT: reduction in the number of adult female ticks, CRW reduction in weight of female ticks CRO: reduction in the egg laying capacity. Efficacy calculation includes significant and non-significant parameters

*Significantly different from control based on Student’s *t* test (*P*  = 0.033)

### Microarray data validation by RT-qPCR

**Fig. 2:**
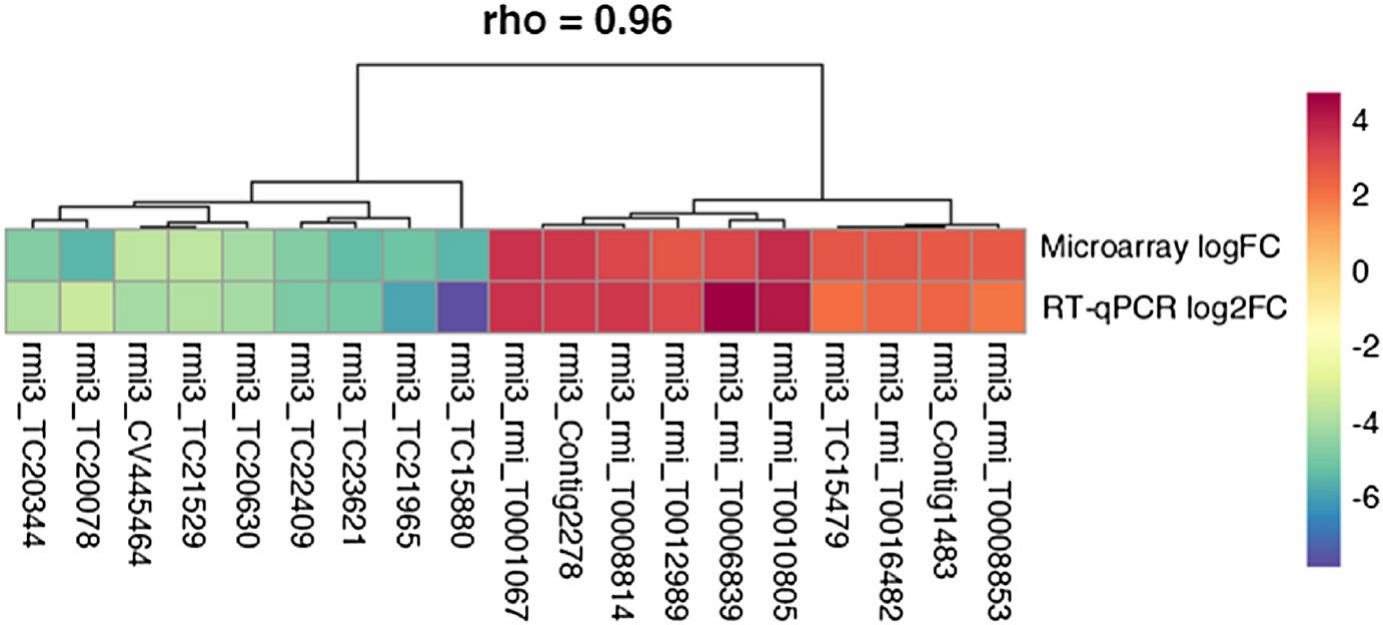
Hierarchical clustering of differentially expressed transcripts based on normalised log_2_FC- and log_2_FC-values obtained from microarray and RT-qPCR analyses, respectively. Each block represents the log_2_FC values of differentially expressed transcripts obtained from microarray analysis (row 1) and RT-qPCR analysis (row 2). Different colour represents difference in expression: Red: increased expression; Blue: decreased expression. Rho: degree of correlation between expression values from DNA microarray- and RT-qPCR analyses determined by Spearman’s rank-order correlation (color figure online)

To validate the microarray data, relative RT-qPCR was performed for ten most upregulated and ten most downregulated transcripts. Analysis of variance (ANOVA) indicated that the expression levels of all 20 transcripts were significantly differentially expressed in the vaccinated group compared to the control group (*P* < 0.05). An overall concordance was observed in the direction and magnitude of fold change values obtained from microarray and RT-qPCR analyses, thereby independently validating the results obtained from the microarray experiment (Fig. 2).

### Functional annotations of differentially expressed transcripts

Blast2GO annotation showed 54% of the differentially expressed transcripts having a significant BLAST hit against the tick annotation release 100, based on the assembly ASM1333972v1 (GCF_013339725.1) (Jia et al. 2020). Some 91% and 83% of the differentially expressed transcripts with significant BLAST hits were assigned GO and InterProScan annotations, respectively.

#### Differentially expressed transcripts involved in digestion of the bloodmeal and vitellogenesis

In ticks, digestion of albumin and haemoglobin from the bloodmeal takes place in the acidic intracellular compartments of the gut epithelium. In the case of haemoglobin, the protein is processed into large fragments by endopeptidases namely: cathepsin D supported by cathepsin L and legumain (Cruz et al. 2010; Fogaça et al. 1999; Sojka et al. 2013). Subsequent proteolysis is mediated by the action of exopeptidases through the dipeptidase activities of cathepsins B and C. It is proposed that monopeptidases, including serine carboxypeptidase and leucine aminopeptidase, participate in the liberation of free amino acids (Cruz et al. 2010; Fogaça et al. 1999; Sojka et al. 2013). As vaccination with Bm86 is known to disrupt digestion and leakage of haemoglobin into the body cavity of ticks, the so called “red phenotype” (Agbede and Kemp 1986), we anticipated the differential expression of numerous proteases and protease inhibitors in ticks fed on vaccinated animals.

Three putative cathepsin D-like transcripts were downregulated in ticks feeding on the vaccinated cattle (T0010166, TC16620 and CV445464), along with a putative legumain-like transcript (TC17290). This highlights disruption of the first steps of digesting haemoglobin into large fragments. In contrast, two putative cathepsin L encoding transcripts (TC15655 and Contig1628) were significantly upregulated (Table 2). Transcript TC15655 has 98% sequence identity to BmCL1 which has been shown to localize to the inside of vesicles within the midgut epithelium of partially engorged females where is it proposed to play a role in blood digestion. However, in a paper by (Xavier et al. 2019) they found that this cathepsin L is the same protein as the previously known BmGTI (*Boophilus microplus* Gut Thrombin Inhibitor). As such, the role of transcript TC15655 may be in both digestion and in mediating anticlotting activity (Renard et al. 2000, 2002; Xavier et al. 2019), and remains to be tested. Lastly, a putative dipeptidyl peptidase 1-like-encoding transcript, also known as Cathepsin C, (Contig1379) was also upregulated in the vaccinated group. This transcript does contain a signal peptide, but it is most likely a lysosome targeting peptide. Targeting this enzyme via vaccination will be challenging due to its intracellular location and being part of a large protein family, which can compensate for loss of function. To date, one multicomponent vaccine containing recombinant tick Cathepsins B, C, D, L and legumain from *I. ricinus* has been evaluated in a rabbit model. Challenge of the vaccinated animals with *I. ricinus* ticks showed a slight decrease of engorged females, despite high antibody titres observed against all the antigens (Franta 2012). The presence of antibodies merely indicate that the proteins are antigenic when administered during vaccination, but as these proteins occur inside the digestive vesicles where they function at acidic pH, they are not accessible to the immune response elicited upon vaccination. The feasibility of vaccinating against intracellular proteins remains questionable and therefore not a priority for further evaluation. Targeting cathepsins using small drugs remains a viable route based on (a) the expansion of novel lysosomal-delivery mechanisms for targeted drug delivery, and (b) rational inhibitor design using protein crystal structures to target parasite specific protein features. This approach is currently exploited for a number of parasites, including *Plasmodium* spp. (Barber et al. 2021; Sojka et al. 2021), Trypanosome spp. (Alvarez et al. 2021) and *Schistosome* spp. (Mughal et al. 2022).

**Table 2:** List of transcripts expressed in adult female ticks fed on vaccinated vs control cattle hosts

General pathway	Contig on array	Description	Putative localization	logFC	Adj. P *
Energy transport and metabolism	T0000903	Putative facilitated glucose transporter, Solute carrier family 2	Extracellular. Cell membrane, multi-pass membrane protein	2.58	5.30E−05
	Contig2278	Putative glucose-6- phosphatase	Intracellular. Endoplasmic reticulum, multi-pass membrane protein	3.93	6.50E−05
Growth and development	TC16526	Putative granulin	Extracellular. Secreted protein	3.20	8.21E−04
Innate immunity	TC23502	Microplusin	Extracellular. Secreted protein	− 3.82	8.20E−04
	CK189663	Putative ixoderin, Techylectin-5A-like	Extracellular. Secreted protein	− 4.00	2.58E−04
	TC22409	Putative ixoderin, Techylectin-5A-like	Extracellular. Secreted protein	− 4.73	6.22E−05
	TC19369	Putative ixoderin, Techylectin-5A-like	Extracellular. Secreted protein	− 3.19	3.60E−04
	AA257910	Putative tubulin alpha-1A chain	Intracellular. Cytoplasm, cytoskeleton	2.83	5.72E−04
	CV444905	Cystatin-L2-like cysteine peptidase inhibitor	Intracellular. Cytoplasm	− 2.49	1.50E−03
Oxidative stress	T0004546	Putative tubulin alpha-1C chain	Intracellular. Cytoplasm, cytoskeleton	2.45	4.47E−04
	F1-2-A_Clone_20	Putative heat shock protein HSP 90-beta	Intracellular and extracellular	1.77	1.57E−03
	T0000364	Putative heat shock protein 105 kDa	Intracellular. Cytoplasm	1.60	2.00E−03
	CK184995	Putative ubiquitin-like modifier-activating enzyme	Intracellular. Cytoplasm (and nucleus)	− 2.76	3.16E−04
	TC24883	Putative hypoxia-inducible prolyl hydroxylase 2	Intracellular. Mainly cytoplasmic	− 3.35	2.91E−04
	TC17253	Putative glutathione S-transferase 1	Intracellular. Cytoplasm	− 3.83	6.11E−04
	TC17897	Putative glutathione S-transferase Mu 2	Intracellular. Cytoplasm	− 3.42	7.80E−04
	TC17316	Putative glutathione S-transferase Mu 2	Intracellular. Cytoplasm	1.72	1.08E−03
	TC16153	Putative methanethiol oxidase, Selenium-binding protein 1-A (alt)	Intracellular. Cytosol and nucleus as peripheral membrane protein	− 2.85	1.84E−04
	TC18726	Putative O-glucosyltransferase rumi homolog	Extracellular. Endoplasmic reticulum, secreted protein	− 2.29	6.38E−04
	TC23300	Putative E3 ubiquitin-protein ligase NEURL1B-like	Intracellular. Cytoplasm	− 3.66	1.59E−04
	NP1774071	Ferritin	Intracellular. Cytoplasm	− 3.50	5.30E−05
Digestion and embryogenesis	TC17290	Legumain-like	Intracellular. Cytoplasm	− 2.70	9.66E−05
	T0010166	Putative cathepsin D-like	Intracellular. Lysosomal membrane	− 2.18	1.20E−03
	TC16620	Putative cathepsin D-like	Intracellular. Lysosomal membrane	− 4.17	3.43E−05
	CV445464	Putative cathepsin D-like	Intracellular. Lysosomal membrane	− 3.58	6.58E−05
	TC15655	Cathepsin L	Intracellular. Lysosomal	1.90	2.31E−03
	Contig1628	Cathepsin L	Intracellular. Lysosomal	2.01	7.67E−04
	Contig1379	Cathepsin C	Intracellular. Lysosomal	1.63	6.24E−03
	TC23222	Vitellogenin-2-like	Extracellular. Secreted protein	− 2.34	3.04E−04
	TC21529	Vitellogenin-2-like	Extracellular. Secreted protein	− 3.59	1.32E−05
	TC21162	Vitellin-degrading cysteine endopeptidase	Extracellular. Secreted protein	− 2.39	1.39E−04
	T0005046	Putative ecdysteroid receptor	Intracellular. Nucleus	2.04	2.77E−04
	TC20218	Niemann Pick type C 1a	Extracellular. Plasma membrane	1.91	3.54E−04
	T0009333	Palmitoyltransferase ZDHHC17-like	Intracellular. Golgi membrane	0.91	1.93E−02
	TC23621	Serine protease inhibitor	Intracellular. Cytoplasm	− 5.57	3.02E−05
	TC20102	Serine protease inhibitor	Intracellular. Cytoplasm	− 4.70	9.75E−05

Indicated are transcripts that are significantly differentially expressed (*P*  < 0.01, logFC ≥ 2 or ≤ − 2). Additional non-differentially expressed transcripts of interest are also indicated with functions within identified pathways

*Adjusted *P* < 0.01 and upper and lower 1% of logFC distribution

One cystatin-L2-like cysteine peptidase inhibitor (CV444905) was downregulated in ticks fed on vaccinated cattle. The role of this inhibitor in *R. microplus* midgut tissues remains unconfirmed. Two serine protease inhibitors (TC23621, TC20102) that belong to the Papilin protein family (and containing Kunitz domains) were downregulated. Their specific biological function(s) remain uncharacterized. All three of these protease inhibitors are predicted to have signal peptides and might be accessible to the immune system. However, all of them are also part of large protein families that may compensate for loss of function during vaccination and/or drug treatment.

In ticks, the process of vitellogenesis is induced by the blood meal and mediated by ecdysteroids (see next section). Vitellin (Vn) is the major yolk protein and serves as an important nutrient for embryonic development. It is processed from its precursor vitellogenin (Vg), a large phosphoglycoprotein produced in the fat body, midgut and ovaries of ticks (Donohue et al. 2008). From these tissues, Vg is released into the hemolymph from where it is taken up by the oocytes via receptor-mediated endocytosis. Once inside the oocytes Vg is converted to Vn (Xavier et al. 2018). Disruption of the uptake of Vg into oocytes via gene known-down studies resulted in reduced fecundity in *R. microplus* ticks, supporting the role of Vg in tick reproduction (Xavier et al. 2018). However, due to vitellogenin’s ability to sequester haem, it might also play a role in haem detoxification, preventing oxidative stress and subsequent tissue damage (Khalil et al. 2013). In ticks fed on vaccinated animals, two vitellogenin-2-like transcripts (TC23222 and TC21529) were significantly decreased in the midgut tissue. Furthermore, a vitellin-degrading cysteine endopeptidase (VTDCE; TC21162) was also significantly downregulated. VTDCE is a cathepsin L-like enzyme fist discovered in the eggs of *R. microplus* (Seixas et al. 2003), but is also localised in the midgut where it is synthesised and transported through the haemolymph to the developing oocytes (Seixas et al. 2010).

These observations are expected as it is known that Bm86 vaccination reduces the fecundity of ticks and disrupts bloodmeal uptake due to leakage into the haemolymph. The question as to whether vaccination against any of the latter transcripts are promising remains to be evaluated, but this approach will face some serious obstacles such as (a) the need for very high antibody titres within the host and the haemolymph of the tick to inhibit the very large number of Vg transcripts, (b) adequate transport of antibodies across the tick midgut barriers in the absence of a barrier disrupting mechanism(s) such as Bm86 vaccination, (c) stability of antibodies within the haemolymph, as IgG has been shown to be rapidly degraded (Benyakir 1989), (d) overcoming the tissue-mediated compensation mechanisms of expressing Vg-transcripts from an array of tissues, (e) antibody binding needs to block the function all VGs to affect tick fecundity, (f) lack of maintaining host immune memory to sustain a protective response, to name but a few. Targeting of the vitellogenin receptor to block receptor-mediated endocytosis of Vg into oocytes remains an option, but will require significant insight into the receptor structure to design tick-specific compounds (Mitchell et al. 2019).

A very interesting gate to parasite control via targeting vitellogenesis came to light in 2021 in a paper by Perdomo et al. (2021). In their study, human blood miRNAs were found to be transported into the fat body tissue of *Aedes aegypti* mosquitoes from where it regulated the expression of mosquito genes. Using artificial feeding with blood containing human miR-21-5p they showed that the miRNA positively regulates the expression of the vitellogenin gene. This study opens the door to targeting host miRNAs (or small regulatory molecules) to disrupt the bloodmeal-mediated activation of parasite gene expression (Perdomo et al. 2021). Also, when combining the expansion of RNA-based therapeutics such as miRNA agomirs (Zhang et al. 2021; Damase et al. 2021), and the increase in studies on the role of regulatory RNAs in parasite fecundity (Li et al. 2021; Zhang and Raikhel 2021) this field holds a lot of promise. It must be noted that insight into the mechanisms regulating gene expression in ticks are essential to fully exploit cutting-edge RNA and/or DNA therapeutics.

#### Ecdysteroid signalling pathway

**Fig. 3:**
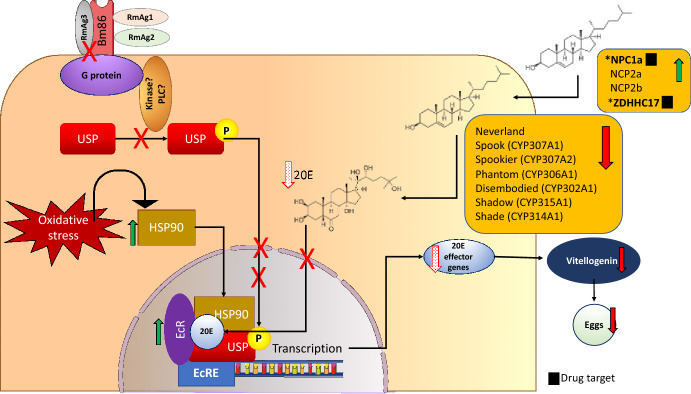
Proposed impact of vaccination on the ecdysteroid pathway in the midgut tissues of female cattle ticks. Ecdysteroid metabolism starts with the uptake of cholesterol and the synthesis of 20E. The enzymes (encoded for by the Halloween genes) needed for the synthesis of 20E is downregulated, and as such the 20E-mediated activation of transcription is suppressed resulting in the lack of expression of 20E effector genes such as vitellogin that impact egg production. Receptors such as the Nieman Pick proteins type C 1a (NPC1a) and a palmitoyltransferase ZDHHC17-like protein involved in lipoprotein transport and protein palmitoylation is upregulated to allow for uptake of cholesterol under stress from vaccination. The cyotoplasmic ecdysteroid receptor (EcR) remains present and does not translocate to the nucleus to activate transcription. To assemble the tranxription machinery, a number of components have been described in the 20E activated transcription complex. We propose that the activation of cytoplasmic components via signaling is disrupted in ticks fed on vaccinated animals. An example of a possible effect on ultraspiracle (USP) is shown

Ecdysteroids and juvenile hormones (JHs) are the two major hormone families that drive development, moulting, and reproductive physiology in insects (Lenaerts et al. 2019). Both hormones mediate their functions via nuclear receptors that control the transcription of an array of genes. Ecdysteroid synthesis, including the active derivative 20-hydroxyecdysone (20E), starts with the uptake of cholesterol. This uptake can be mediated by cholesterol transporters such as Nieman Pick proteins (Xavier et al. 2021) or via lipoproteins that enter cells through receptor-mediated endocytosis (Miller and Bose 2011). Cholesterol is converted enzymatically to 7-dehydrocholesterol by the oxygenase enzyme neverland (nvd). The remainder of the ecdysteroidogenic pathway is mediated by a number of cytochrome P450 enzymes (a.k.a. the Halloween genes), which mediates a series of oxidation and hydroxylation steps to finally produce 20E. These enzymes include: Cyp307a1/spook (spo); Cyp307a2/spookier (spok); Cyp306a1/phantom (phm); Cyp302a1/disembodied (dib); Cyp315a1/shadow (sad); and Cyp314a1/shade (shd) (Niwa and Niwa 2016). Once 20E is produced, it acts via the heterodimeric ecdysone receptor complex, ecdysone receptor/ultraspiracle (EcR/USP), to affect gene expression. In 2012, co-localisation studies in *Rhodnius prolixus* cells furthermore indicated that the EcR co-localises with Hsp90, the immunophilin FKBP52, the light chain 1 of the motor protein dynein and microtubules (Vafopoulou and Steel 2012), all of which was deemed essential for the intact nucleocytoplasmic shuttling of EcR (Fig. 3). Under conditions that cause depolymerization of microtubules, there was a clear reduction in nuclear EcR and a concomitant increase in cytoplasmic EcR (Vafopoulou and Steel 2012). In 2013 Liu et al*.* showed that also in *Helicoverpa armigera* (Lepidoptera) the ultraspiracle protein interacts with Hsp90 and proposed the formation of the transcription complex containing 20E, EcR, USP1 (phosphorylated) and Hsp90 that recognise ecdysone response elements (Liu et al. 2013) (Fig. 3). In *Ornithodoros moubata* (Horigane et al. 2008, 2007; Connat et al. 1984; Ogihara et al. 2015), *Amblyomma americanum* (Palmer et al. 2002), and *Haemaphysalis longicornis* (Yang and Liu 2022) ticks, it has been shown that the ecdysteroid pathway is induced by blood feeding and that 20E is vital to tick fitness.

It must be noted that in insects, ecdysteroid synthesis occurs in a specialised endocrine organ called the prothoracic gland which ticks do not possess. Instead, in both argasid and ixodid ticks, ecdysone is released from epidermal cells from where they enter haemolymph and affect a number of other tissues/organs such as the ovaries (Reuben Kaufman 2007).

In this study, whole midgut tissue was dissected and used for RNA isolation, and as such the tissue would have contained epidermal cells along with other smaller structures associated with the midgut. From the differentially expressed gene information obtained, a putative Niemann Pick type C 1a (NPC1a) (TC20218) was found as upregulated. Niemann-Pick type C1 is a large membrane glycoprotein with mostly a late endosomal localization which functions in the processing and utilization of endocytosed cholesterol as well as intracellular regulation of cholesterol metabolism (Hu et al. 2020). Another transcript (T0009333) encoding a palmitoyltransferase ZDHHC17-like protein involved in lipoprotein transport and protein palmitoylation was also upregulated. As it is known that ticks require cholesterol and lipids from their bloodmeal, it is expected that proteins involved in cholesterol and lipid uptake is upregulated. With regards to the Halloween genes: Neverland, Phantom, Disembodied and Shadow was found as downregulated in ticks fed on vaccinated animals, but the statistical significance of the data is low. For Spook and Shade very low gene expression levels was found in ticks fed on vaccinated animals. As such, the expression of these genes need to be further analysed using qPCR and the levels of 20E determined in future. A transcript encoding a putative ecdysteroid receptor (EcR) (T0005046) and a putative chaperone Hsp90 (F1-2-A_Clone_20) was significantly upregulated in ticks that fed on vaccinated cattle. No data for USP were found. Based on (a) the downregulation of the Halloween Genes and associated 20E synthesis, and (b) severe disruption of the cytoskeleton as discussed below and (c) the downregulation of vitellogenin which is a 20E response element regulated gene (Elgendy et al. 2021; Zhu et al. 2007), we propose that the EcR remains cytoplasmic and does not translocate to the nucleus to activate transcription (Fig. 3). This model needs to be verified, as it may pave the way forward to understanding the cellular function of Bm86. Taking this work further, it is evident that targeting cholesterol metabolism, and specifically the Niemann Pick type protein(s) are promising drug targets (Fig. 3). This approach is gaining attention in parasite control, especially in Plasmodium (Istvan et al. 2019; Ressurreição and van Ooij 2021), ticks (Xavier et al. 2021; Cabezas-Cruz et al. 2019; Marchesini et al. 2021) and mites (Mani et al. 2022).

#### Tick energy metabolism

**Fig. 4:**
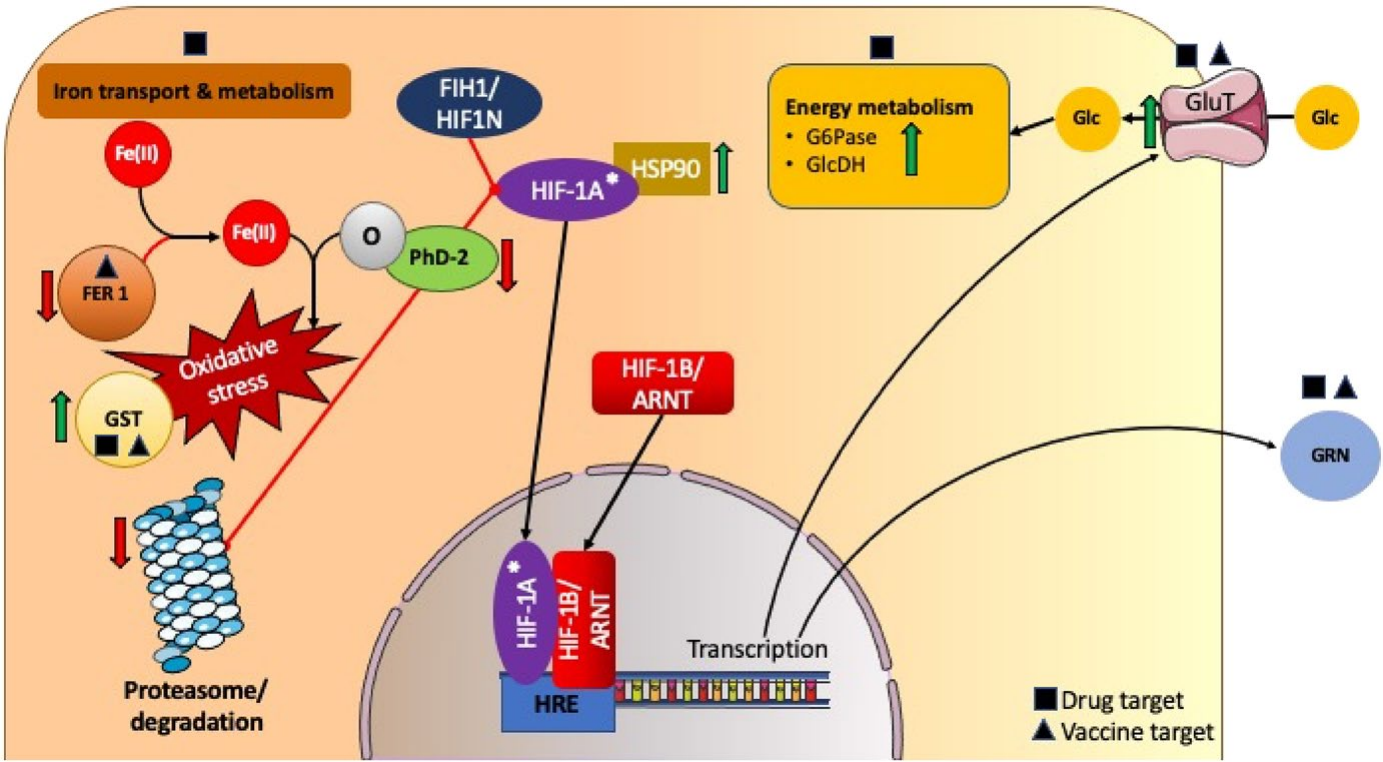
Proposed pathway induced by oxidative stress in the midgut tissues of female cattle ticks feeding on vaccinated cattle hosts. Since important iron transport proteins (i.e., Ferritin-1 or FER-1) are down-regulated in the midguts of ticks feeding on vaccinated hosts, an increasing hypoxic environment results as oxygen is depleted by free ferric iron. Depleting oxygen levels causes the inhibition of a hypoxia-inducible prolyl hydroxylase 2 (PHD-2) enzyme, as well as a concomitant decrease in ubiquitin-mediated proteasomal degradation of the transcriptional regulator Hypoxia-inducible factor-1 alpha (HIF-1). Stabilized by Heat shock protein 90 (HSP90), HIF-1A translocates to the nucleus where it forms a bi-functional regulator with its beta subunit HIF-1B that binds to chromosomal hypoxia-induced response elements (HRE) to initiate transcription of targets that promote cellular adaptation to oxidative stress and hypoxia that include energy metabolism (e.g. enzymes and transporters) and cell survival (e.g. growth factors such as granulin or GRN). Asterisks indicates proteins that were identified in the transcriptome, but was not significantly differentially expressed. Green and red arrows indicate transcripts that were identified to be significantly up– and down-regulated, respectively. Targets for development of novel antiparasitic chemotherapeutics and vaccine targets are indicated by black squares and triangles, respectively (color figure online)

Ingestion of anti-Bm86 antibodies by feeding ticks, results in antibody binding to the midgut luminal surfaces, and in conjunction with complement, results in an apparent lysis of gut epithelial cells and leakage of the gut contents (Willadsen 1997). As the primary organ for blood meal digestion and nutrient acquisition, disruption of the integrity and normal functioning of the midgut tissues will have a ‘knock-on’ effect on normal metabolic processes, possibly leading to dysfunctional nutrient acquisition and depletion of energy stores (Fig. 4). In this study, the apparent effect of this disruption is at least partially supported by the decreased expression of transcripts encoding proteins involved in blood meal digestion (e.g., proteases and peptidases) and nutrient transport (e.g., ferritin) (Table 2). In energy metabolism, dietary glucose (stored as glycogen) is a primary source for immediate energy for both ixodid and argasid ticks, whereas lipids (stored as triglycerides) also have other roles including structural components (Alasmari and Wall 2021; Cabezas-Cruz et al. 2017; Oleaga et al. 2017).

As expected, several transcripts with putative functions in both carbohydrate and lipid transport and metabolism were differentially expressed in female ticks fed on vaccinated cattle (relative to controls) (Table 2). Two of these transcripts encoded proteins that were significantly upregulated in female ticks feeding on vaccinated cattle (logFC > 2), and included a putative glucose-6-phosphatase (Contig2278), as well as a putative glucose transporter of the solute carrier family 2 (T0000903). It is hypothesized that transcripts such as these may function as part of a compensatory or rescue mechanism, to obtain dietary glucose for down-stream metabolic processes. In regards to targeting such transcripts for tick vaccine development, there is unfortunately no data currently available. A peptide vaccine designed against a similar *Anopheles stephensi* glucose transporter (GLUT1), was able to reduce mosquito survival by 5% in vaccinated mice (Couto et al. 2017). To further illustrate the hurdle in vaccine development within this space, only a DNA vaccine directed against a *Schistosoma japonicum* triose-phosphate isomerase (an enzyme traditionally involved in gluconeogenesis), was tested in pigs that resulted in a significant ~ 48% reduction in adult worm burdens in vaccinated pigs (Zhu et al. 2006). Moreover, this mostly intracellular protein was shown to also be secreted to the surface of the fluke (Jimenez-Sandoval et al. 2020), therefore, enabling it to elicit an effective host antibody response. For *R. microplus, *in vitro tick triose-phosphate isomerase (rRmTIM) inhibition assays using murine-derived monoclonal antibodies, demonstrated a high degree of enzyme inhibition (> 48%) (Saramago et al. 2012). But no in vivo vaccine efficacy in animal infestation trials have been demonstrated to date to validate selection of such a target for vaccine development.

In contrast, current research appears to focus more on targeting transporters and other intracellular enzymes involved in energy metabolism for chemotherapeutic control, e.g., to block transmission of parasitic protists such as *Plasmodium* (Wang and Wang 2020), *Trypanosoma* spp. (Haanstra et al. 2017) and *Leishmania* spp. (Ortiz et al. 2017). Such enzymes are also being explored as targets for drug development against helminths (Jimenez-Sandoval et al. 2020), e.g., *Schistosoma mansoni* (Hulme et al. 2022). Therefore, transcripts identified in this study that are related to energy metabolism are considered poor targets for vaccine development, due to the intracellular origin of most of these targets and the concomitant poor extracellular exposure for host immune interactions to confer protection. However, results may still offer targets for small drug development as next generation acaricides.

#### Oxidative stress responses

Blood feeding and digestion is also a source of oxidative stress since free haem and iron, as well as other molecules, that are released following haemolysis can be toxic to the tick tissues (Sabadin et al. 2021; Citelli et al. 2007). Therefore, ticks should have a well-orchestrated oxidative stress response to protect themselves from the deleterious effects of these by-products (Paes et al. 2001; Graca-Souza et al. 2006; Citelli et al. 2007).

Free cytoplasmic ferric iron released during heme digestion is sequestered intracellularly by a class of storage protein, ferritin 1 (Fer1), whereas iron destined for peripheral tissues are transported from digestive cells throughout the hemolymph bound to a secreted ferritin 2 (Fer2) (Kopáček et al. 2018). A putative ferritin 1 (NP1774071) was downregulated in tick midguts that fed on the vaccinated test group (Table 2). Since no other similar proteins were up-regulated it could be hypothesized that the midgut cells were experiencing a buildup of cytoplasmic iron (i.e., iron overload), similar to findings from knock-down experiments in other tick species (e.g. *Haemaphysalis longicornis*) (Galay et al. 2014b). The ferritin 2 protein has been tested as a vaccine target, and homologous challenge trials in cattle showed a 64% and 72% vaccine efficacy against infestations of *R. microplus* and *R. annulatus* ticks, respectively (Hajdusek et al. 2010). The use of ferritin 1 as a vaccine antigen has only been demonstrated in a rabbit model against *H. longicornis* infestation with an efficacy of ~ 34% (Galay et al. 2014a). However, since both tick ferritin 1 and 2 share a high degree of identity, the efficacy observed may be due to antibody cross-reactivity and not specific reactivity towards the intracellular ferritin 1. Iron metabolism as a whole is yet to be explored for chemotherapeutics, as has been the case for various human disorders (Crielaard et al. 2017).

Several transcripts involved with the glutathione detoxification pathway (TC17253 and TC17897) were also significantly down-regulated (Table 2). Glutathione S-transferase is a known phase II detoxification enzyme involved in management of oxidative stress. These enzymes are highly expressed in tick tissues during tick feeding where they act as intracellular scavengers of free heme (Rodriguez-Valle et al. 2010; Kopáček et al. 2018), similarly to other blood-feeding parasites, including *Haemonchus contortus* (van Rossum et al. 2004) and *Ancylostoma caninum* (Zhan et al. 2005). Therefore, down-regulation of these proteins can support the notion that the midgut tissues of ticks feeding on the test group were experiencing increased oxidative stress. A compensation mechanism is, however, evident as a putative GST encoding transcript (TC17316) was significantly upregulated (Table 2). A study by Sabadin et al. (2017) showed > 80% sequence similarity of a GST sequence from *H. longicornis* ticks (GST-HI) to GST from *R. appendiculatus* and *R. sanguineus*, with multiple conserved antigenic sites. Vaccination with recombinant GST from *H. longicornis* showed 57% efficacy against *R. microplus* infestation, showing anti-GST cross-reactivity in both species (Parizi et al. 2011). Consequently, the product of this transcript could be considered for further investigation as an additional candidate for a multivalent vaccine.

Hypoxia-inducible factor (HIF)-1 is a key transcriptional regulator that promotes cellular adaptation to oxidative stress and hypoxia for key biological processes that include energy metabolism, apoptosis and cell survival (Movafagh et al. 2015). A putative Egl nine homolog 1 transcript (TC24883) encoding a putative hypoxia-inducible prolyl hydroxylase 2 (PHD-2) was significantly downregulated (logFC > 3) in the test group (Table 2). This enzyme hydroxylates a proline residue in the alpha subunit of the HIF-1 transcription factor to enable proteasomal destruction by the ubiquitin E3 ligase complex under normal oxygen tension (Jaakkola et al. 2001), whereas enzyme function is inhibited by low oxygen concentrations or hypoxia (Stowe and Camara 2009). As a result of an increase in pro-oxidants (e.g., Fe(II) and heme), a hypoxic environment could be created within the tick midgut tissues that inhibits the function of PHD-2. Consequently, HIF-1 alpha is not degraded by the proteasome following ubiquitination and therefore proceeds to form a transcription complex with the HIF-1 beta subunit which then acts as a master regulator of numerous hypoxia-inducible genes (Greer et al. 2012). This observation is further substantiated by the concomitant decreased abundance of transcripts (i.e., CK184995) that have putative functions in ubiquitination and perhaps degradation of HIF-1 alpha (Kamura et al. 2000; Tandle et al. 2009). For additional support, a putative selenium-binding protein (TC16153) was also down-regulated that has been implicated to be a negative regulator of HIF-1 alpha during recovery from injury and cancer progression in humans (Jeong et al. 2014; Seelig et al. 2021). However, this function has not been described in arthropods to date and can be tested further in future studies.

Transcripts encoding the HIF-1 regulator molecule were identified in this study to be non-differentially expressed (data not shown), this is expected as it has been shown that HIF-1 in humans is constitutively expressed (Ke and Costa, 2006). Increased levels of Hsp90 and Hsp70 (Genin et al. 2008), stabilises HIF-1 alpha and protects it from degradation by the proteasome (Zhou et al. 2004). Putative Hsp70 (T0000364) and Hsp90 (F1-2-A_Clone_20) encoding transcripts were upregulated in ticks feeding on vaccinated hosts. The inhibition of a putative PHD-2 under hypoxic conditions has also been associated with cytoskeletal remodelling assisting in translocation of HIF-1 alpha to the nucleus, where it can associate with HIF-1 beta to form the functional transcription factor (Guo et al. 2017; Weidemann et al. 2013). In this regard, two putative alpha-tubulin encoding transcripts (T0004546 and AA257910) were significantly upregulated (logFC > 2) (Table 2). In response to oxygen-depletion, HIF-1 signalling can regulate glucose uptake and anaerobic responses by activating the transcription of glucose transporter 1 (GLUT1) (Chen et al. 2001), as shown by the upregulation of a GLUT1 encoding transcript (T0000903) (Table 2).

Recent evidence also suggests that hypoxic stress can suppress Notch activity, involved in cell-to-cell communication during tissue development, and expression of its downstream signalling molecules (Itoh et al. 2019). In this regard, two transcripts encoding putative proteins involved in the Notch signalling pathway were downregulated in this study (i.e., TC23300 and TC18726) (Table 2). Consequently, an alternative pathway to promote cell growth and differentiation may be up-regulated. This hypothesis is based on the up-regulation of a putative epithelial growth factor, granulin (TC16526), which has been shown in mice to induce DNA synthesis and stimulate cell growth by activating mitogen-activating protein kinase (MAPK) and the phosphatidylinositol 3-kinase (PI-3 K) pathways (Zanocco-Marani et al. 1999). Activation of these pathways lead to cell growth and activation of crucial metabolic functions such as synthesis of lipids, proteins and glycogen (Lodish et al. 2008).

Taken together, the data identifies possible metabolic pathways that serve as a rescue mechanism explaining why ticks can survive even after vaccination, which disrupts feeding and growth. Only a granulin-like growth factor (Ov-GRN-1) from the fluke *Opisthorchis viverrini* has been considered for further development as a potential vaccine candidate (McManus 2020). However, the utility of this target as a tick vaccine antigen remains to be demonstrated. Intracelluar targets such as ubiquitin (UBQ) and elongation factor 1 alpha (EF1a), have been tested in cattle vaccination and *R. microplus* infestation trials, with 0% and 38% vaccine efficacies reported, respectively (Almazán et al. 2012). No other targets of the HIF-1 alpha pathway have been successfully exploited for antiparasitic vaccine development and since the majority of these targets are intracellular, these may rather be pursued as targets for future drug development.

#### Innate immune system

During parasite feeding, ticks can be subjected to pathogens in the host’s blood and/or which reside in the tick gut (van Oosterwijk and Wikel 2021). Several transcripts that have a putative role in the tick immune system were significantly downregulated (logFC > 2) in the midgut tissue of ticks that fed on vaccinated cattle. These include putative ixoderins (CK189663, TC22409 and TC19369) and the antimicrobial peptide microplusin (TC23502) (Table 2). The continuing co-evolution of ticks with their associated pathogens promotes favourable characteristics of tick physiology to successfully maintain and transmit pathogens without compromising the fitness of the tick vector (Mans 2011). Ticks feeding on vaccinated cattle seem to also have a compromised innate immunity, and it is hypothesised that the Bm86 and RmAg3 proteins may function synergistically in vivo to protect the tick from the damaging effects of its ingested microbes. Disruption of the innate immune system of ticks could potentially prevent the successful colonisation and eventual transmission of microbes to the host and also represents a target for transmission blocking vaccines (van Oosterwijk and Wikel 2021). This has in principle been proven for cattle vaccinated with Bm86, which reduced the transmission of *Anaplasma marginale* by reducing the number of infected ticks (de la Fuente et al. 1998). However, targeting tick innate immunity per se for tick control, remains to be validated as no vaccines successfully targeting this aspect of tick biology exist. Similarly, targeting innate immunity for antiparasitic drugs has also not been demonstrated to date. However, tick-derived antimicrobial peptides have been explored as treatments for human microbial infections, e.g., microplusin against *Cryptococcus neoformans* (Silva et al. 2011).

## Conclusions

Multicomponent vaccines have enjoyed a lot of attention over the past two decades in the development of vaccines against complex parasites. Examples include, but are not limited to vaccines against parasitic worms such as the nematode *Teladorsagia circumcincta* (Nisbet et al. 2013), tapeworms (*Taenia saginata)* (Lightowlers et al. 1996), mites (*Dermanyssus gallinae*) (Bartley et al. 2012) and ticks (Ndawula and Tabor 2020). Multicomponent vaccines that target the various life stages of *Plasmodium falciparum* is also well documented such as vaccines that target multiple steps in the erythrocyte invasion pathway (Bustamante et al. 2017), multiple developmental stages (Boes et al. 2015), and protein–protein interactions (PPIs) within *P. yoelli* (Spring et al. 2009; Ouattara et al. 2010; Thera et al. 2011). The development of multicomponent vaccines for ticks will require an in-depth understanding of tick biology and the complex interaction of responses that occur during the whole life cycle, and in response to current vaccines on the market. Criteria for the selection of multicomponent vaccines following expression profiling remains challenging, as insight into the effects of concentration of antigen expression; timing and length of expression in the parasite and subcellular location, remains unknown. Clearly there are many questions to deal with in this area.

To date, vaccines against complex parasites fail to offer complete protection, and as such the combination of vaccines with small drugs (anti-parasitic drugs and acaricides) remains at the forefront. With the increase in acaricide resistance, new targets for chemical control is in dire need. This include the development of new acaricides and/or small drugs. Development of new therapeutics is also attractive as small drugs can potentially target numerous tick species, and have potentially lower production costs. High-throughput in silico drug discovery can be attempted, even in the absence of crystal structure data. Tools such as AlphaFold (Jumper et al. 2021) will enable ab initio prediction of high quality target protein structure models that can be used in drug docking studies. In summary, this paper provides a number of targets for the future development of vaccines and small molecular therapeutics, as well as novel pathways that can be targeted towards improving the outcome on tick control.

### Supplementary Information

Below is the link to the electronic supplementary material.

Supplementary file1 (DOCX 16 KB)

## Data Availability

Normalized microarray data was submitted to the GEO repository of the National Centre for Biotechnology Information on accession no GSE221646.
