# Supplementary material for: Mining gene expression data for rational identification of novel drug targets and vaccine candidates against the cattle tick, Rhipicephalus microplus
Source: Exp Appl Acarol. 2023 Sep 27;91(2):291–317. doi: 10.1007/s10493-023-00838-8 (PMC10562289; doi:10.1007/s10493-023-00838-8)
Supplement: Supplementary file 1 — Supplementary file1 (DOCX 16 KB) [file 10493_2023_838_MOESM1_ESM.docx]

**SUPPLEMENTARY DATA**

**Supplementary Table 1: Primers used for qPCR validation.**

| **Transcript Name** | **Sequence description** | **Forward Primer 5'-3'** | **Reverse primer 5'-3'** | **Product length** |
| --- | --- | --- | --- | --- |
| rmi3_Contig2278 | Glucose-6- phosphatase | GTGATGCCAAATCGTCCC | AATGTCACGCTGTTTAACGAA | 128 |
| rmi3_rmi_T0010805 | Antigen b membrane protein | ACATTCTGGATACCGCTGTCG | CTCGTTCAAGTCAGTCACCGTA | 140 |
| rmi3_rmi_T0001067 | Apical endosomal glycoprotein | GCCCATCTCCTCAAGAAATAG | TCGCTGAACTCAATGTCGTC | 105 |
| rmi3_rmi_T0016482 | Antigen b membrane protein | GACGACAACAGCATCCACG | CATCTTATCACCCGGCACGAA | 102 |
| rmi3_rmi_T0006839 | Pr domain zinc finger protein 1 | TCAAGCCATCGCAGACGCTCA | CCGTCGTACACTCGCCAGA | 157 |
| rmi3_rmi_T0008814 | Tick serine proteinase | GCCAGAATACTCCCTCAACGAC | TCACCATCTTCGTCTCGGTCA | 90 |
| rmi3_rmi_T0008853 | Lipase | GCCCGGACTGTTCCCATTCTCG | CGAAGCATTGTAGTCGGCGCAC | 98 |
| rmi3_TC15479 | Antigen B membrane protein | TCTCGCCTCTTCGTATCCCAA | ATCTTATCACCCGGCACGAAC | 142 |
| rmi3_rmi_T0012989 | Antigen B membrane protein | CCTATGCCTTTGTCAACCAC | CGCAATAAATAGAAACAACGAAGC | 155 |
| rmi3_Contig1483 | Basement membrane-specific heparan sulfate proteoglycan core | CAGCCAAGTGAAAACATCGG | TTGATGATGCTCGATACCTGA | 128 |
| rmi3_TC23621 | Serine proteinase inhibitor | TGTCAGAGCAACGGCAACAAC | TCACCGAGACTTCAAGAGC | 86 |
| rmi3_TC20078 | Serine proteinase inhibitor | TTTTACAACACCGAGACGAAG | GCCGAAAACCTTCAGACAC | 117 |
| rmi3_TC15880 | Cathepsin D | CCCATCCCGATAATACTCACC | CAATTAATCGACGGCACCCA | 134 |
| rmi3_TC21965 | Secreted salivary gland protein | TTTGAGCAATGTACGCCCAC | CTTTGACACACCATGAGCC | 85 |
| rmi3_TC20344 | Insulin-like growth factor binding protein | CTGCCCATTGAATTCCC | TTTCTCGACAGGTAAATGGC | 130 |
| rmi3_TC22409 | Ixoderin | TGCGAATCGTATTGAGCAAC | TCCCGTAAAATCTCCTATCCG | 113 |
| rmi3_TC21557 | Calcium-dependent cysteine protease | GTACGACAAAGGCTCAACC | GATTGACCCTGTATCCTGC | 80 |
| rmi3_TC20630 | Insulin-like growth factor binding protein | ATGTCCCAACGAAGAGTGCAA | TGCCTCCGTTCTCAAAGCG | 101 |
| rmi3_TC21529 | GP80 (vitellogenin) | TCACCAACTCGCTCAAGA | GACACAAGCCAGTTCTCG | 157 |
| rmi3_CV445464 | Aspartic protease | GGACGCTCAATATTACGGCAAC | CACACTCTCTGGCGGACA | 121 |
| **Reference Gene** |  | **Forward Primer 5'-3'** | **Reverse primer 5'-3'** | **Product length** |
| ELF1α | Elongation factor 1-alpha | CGTCTACAAGATTGGTGGCATT | CTCAGTGGTCAGGTTGGCAG | 108 |
| RPL4 | Ribosomal protein L4 | AGGTTCCCCTGGTGGTGAG | GTTCCTCATCTTTCCCTTGCC | 152 |
